# Surveillance of *Aedes aegypti* populations in the city of Praia, Cape Verde: Zika virus infection, insecticide resistance and genetic diversity

**DOI:** 10.1186/s13071-020-04356-z

**Published:** 2020-09-21

**Authors:** Monica Campos, Daniel Ward, Raika Francesca Morales, Ana Rita Gomes, Keily Silva, Nuno Sepúlveda, Lara Ferrero Gomez, Taane G. Clark, Susana Campino

**Affiliations:** 1 Faculty of Infectious and Tropical Diseases, London School of Hygiene and Tropical Medicine, London, UK; 2 Laboratory of Pathogen-Host Interactions (LPHI), UMR5235, CNRS, Montpellier University, 34095 Montpellier, France; 3 Universidade Jean Piaget (UniPiaget), Praia, Cabo Verde; 4 Centre of Statistics and Its Applications of University of Lisbon, Lisbon, Portugal; 5 Faculty of Epidemiology and Population Health, London School of Hygiene and Tropical Medicine, London, UK

**Keywords:** *Aedes aegypt*i, Zika, Cape Verde, *kdr*, *nad*4

## Abstract

**Background:**

*Aedes* spp. are responsible for the transmission of many arboviruses, which contribute to rising human morbidity and mortality worldwide. The *Aedes aegypti* mosquito is a main vector for chikungunya, dengue and yellow fever infections, whose incidence have been increasing and distribution expanding. This vector has also driven the emergence of the Zika virus (ZIKV), first reported in Africa which spread rapidly to Asia and more recently across the Americas. During the outbreak in the Americas, Cape Verde became the first African country declaring a Zika epidemic, with confirmed cases of microcephaly. Here we investigate the prevalence of ZIKV and dengue (DENV) infected *Ae. aegypti* mosquitoes in the weeks following the outbreak in Cape Verde, and the presence of insecticide resistance in the circulating vector population. Genetic diversity in the mosquito population was also analysed.

**Methods:**

From August to October 2016, 816 *Ae. aegypti* mosquitoes were collected in several locations across Praia, Cape Verde, the major hot spot of reported ZIKV cases in the country. All mosquitoes were screened by reverse transcription PCR for ZIKV and DENV, and a subset (*n* = 220) were screened for knockdown insecticide resistance associated mutations in the voltage gated sodium channel (*VGSC*) gene by capillary sequencing. The mitochondrial *NADH dehydrogenase subunit 4* (*nad*4) gene was sequenced in 100 mosquitoes. These data were compared to 977 global sequences in a haplotype network and a phylogenetic tree analysis.

**Results:**

Two *Ae. aegypti* mosquitoes were ZIKV positive (0.25%). There were no SNP mutations found in the *VGSC* gene associated with insecticide resistance. Analysis of the *nad*4 gene revealed 11 haplotypes in the Cape Verdean samples, with 5 being singletons. Seven haplotypes were exclusive to Cape Verde. Several of the remaining haplotypes were frequent in the global dataset, being present in several countries (including Cape Verde) across five different continents. The most common haplotype in Cape Verde (50.6 %) was also found in Africa and South America.

**Conclusions:**

There was low-level Zika virus circulation in mosquitoes from Praia shortly after the outbreak. The *Ae. aegypti* population did not appear to have the *kdr* mutations associated with pyrethroid resistance. Furthermore, haplotype and phylogenetic analyses revealed that Cape Verde *Ae. aegypti* mosquitoes are most closely related to those from other countries in Africa and South America.
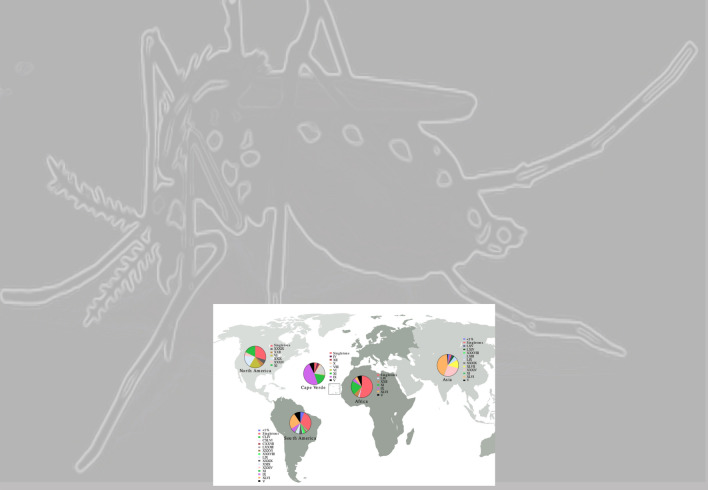

## Background

In recent decades, there has been a rise in the emergence and re-emergence of epidemic arboviral diseases, including those caused by yellow fever (YFV), dengue (DENV), chikungunya (CHIKV) and Zika (ZIKV) viruses [1–3]. More than 2.5 billion people in over 100 countries are at risk of contracting dengue [4], and the Asian strain of the ZIKV has spread throughout the Americas [5–7]. CHIKV has also reached the Americas and has undergone recent rapid spread [8, 9]. Outbreaks of yellow fever in unvaccinated individuals have been reported in the Americas and in Africa and there is a risk it is imported into Asia [10]. The *Aedes aegypti* mosquito is the main vector responsible for the transmission of DENV, CHIKV, YFV and ZIKV worldwide due to its highly anthropophilic behaviour and close proximity with the human environment [11]. This mosquito species thrives in both fresh and stagnated water, in vessels ranging from barrels to bottle caps, and it colonises human residencies. An anthropophilic preference to feeding on human blood and a tendency to feed multiple times during an egg-laying cycle, imparts this particular vector with a remarkable efficiency in pathogen transmission [12–14]. *Aedes aegypti* mosquitoes are currently found in 188 countries and territories, putting an estimated 3 billion people at risk of the aforementioned and future-emerging arboviral diseases [15].

Cape Verde is an archipelago in West Africa comprising of ten volcanic islands located 550 km west from Senegal. *Aedes aegypti* mosquitoes were first detected in 1931 on the island of São Vicente and subsequently spread to the other islands [16, 17]. There are no records of other species of *Aedes* vector, such as *Aedes albopictus* in the region, nevertheless the presence of the non-vector species such as *Aedes caspius* has been recorded [17]. Mitochondrial DNA sequencing analysis of *Ae. aegypti* mosquitoes from Africa indicated a possible West African origin of the Cape Verdean population [18]. With heavy human and goods trans-Atlantic traffic coming in and out of São Vicente, particularly during the 16th to 17th centuries, it is possible that the *Ae. aegypti* population from Cape Verde was imported from regions of the West African coast and also contributed to the New World population. Mitochondrial DNA sequencing analysis among mosquito populations from different geographical locations is a well-described method of determining mosquito ancestry, as well as for the analysis of genetic diversity [19–21]. With the increase in international travel, there is a latent threat that new strains of arboviruses or new vectors are introduced worldwide, and it is important to study the population genetic diversity of *Ae. aegypti*. Cape Verde continues to be a strategic trans-Atlantic route linking particularly West Africa countries with Europe and the Americas [22].

The first arboviral outbreak in Cape Verde occurred in 2009, most likely originating from neighbouring countries in West Africa [23]. This outbreak resulted in 21,137 reported DENV suspected cases and four registered deaths. Eight out of the nine inhabited islands in the archipelago were affected, with Santiago Island, where the city capital of Praia is located, reporting the greatest number of cases [21]. *Aedes aegypti* was identified as the vector, where DENV-3 and DENV-4 virus strains were detected in mosquitoes from Cape Verde and Senegal [23, 24]. A second arboviral outbreak in Cape Verde, caused by ZIKV, occurred from October 2015 to July 2016, shortly following its establishment in Brazil, where the first congenital ZIKV microcephaly cases were reported. The Cape Verdean Ministry of Health officially declared the Zika virus epidemic on 2 November 2015, becoming the first African country to register an epidemic for this virus. There were 7589 suspected cases of ZIKV infection and 18 microcephaly cases officially recorded in Cape Verde, making it the first African country to report ZIKV-associated microcephaly cases [25]. It is possible that the outbreak was caused by importation of strains circulating in the Americas, given the comparable clinical consequences, timing and traveling from those regions [25].

Following the DENV and ZIKV outbreaks in Cape Verde, vector control measures were reinforced which included public education efforts and use of the insecticides temephos (organophosphate) and deltamethrin (pyrethroid), targeting larva and adult mosquitoes, respectively, as well as adulterated diesel and the mosquito fish *Gambusia* sp. for larval control [26].

Pyrethroids, the primary choice of control against adult *Ae. aegypti,* target the voltage gated sodium channel (VGSC) [27]. Knockdown resistance (*kdr*) occurs when an amino acid substitution on the *VGSC* gene reduces the binding affinity of the pyrethroids. The evolutionary selection of mutations in the *VGSC* gene that confer pyrethroid resistance have been described [27]. F1534C is the most common *kdr* mutation, detected in Asia, Africa and the Americas [28]. Several other mutations have been reported, including the V1016G mutation found in Asia [29], and V1016I particularly detected in Latin America and Africa [30]. Co-mutation confers higher levels of resistance, such as the triple mutations of F1534C/V1016I/ S989P that confer extreme resistance, as detected in Myanmar [31]. These mutations have not been detected in *Ae. aegypti* collected in Cape Verde between 2007 and 2014 [18, 32, 33]. However, insecticide susceptibility assays have revealed resistance to dichlorodiphenyltrichloroethane (DDT), the carbamate propoxur, pyrethroids (deltamethrin, cypermethrin) and to the organophosphate temephos [32, 33].

With ongoing issues surrounding the deployment of certain *Flavivirus* vaccines and ineffective antiviral treatment [34], the prevention and control of ZIKV and DENV diseases rely on ongoing surveillance of arboviruses in mosquito vectors and vector control. While insecticides are still the primary measure used for vector control, it is crucial to identify acquired insecticide resistance at an early stage for control efforts to remain effective. Here we aimed to perform the surveillance in *Ae. aegypti* mosquitoes collected in Praia, Cape Verde, just after the ZIKV outbreak in 2015, investigating the presence of ZIKV and DENV infections and screening for *kdr* mutations. In addition, we also assessed the local genetic diversity, phylogeny and ancestry of the *Ae. aegypti* mosquito population by analysing mitochondrial sequences of the *nad*4 gene and comparing them with a global dataset.

## Methods

### Field-collected mosquitoes

**Fig. 1:**
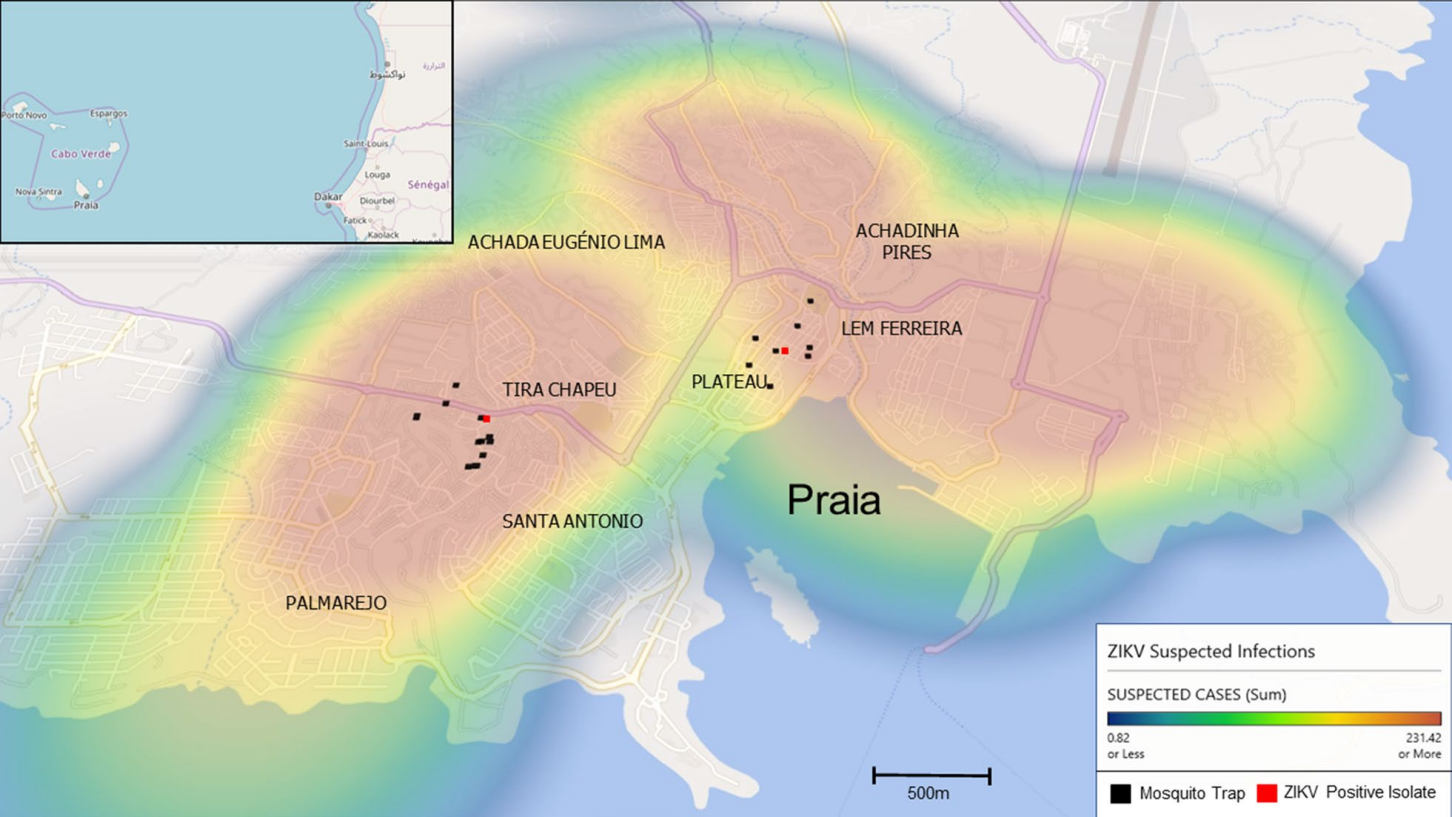
Map showing mosquitoes sampling sites in Praia city, Santiago Island. Sample sites are indicated by black points. Sampling sites with ZIKV positive mosquitoes are indicated in red. Heat map indicates the number of ZIKV suspected infections in a given area

A total of 816 *Ae. aegypti* adult mosquitoes were collected across 27 locations in the capital city of Praia located in Santiago Island, Cape Verde, for seven weeks from 17 August to 5 October 2016 (Fig. 1). The locations were centered in two areas: Tira Chapeu (*n* = 309 mosquitoes, within 500 m of GPS coordinates 14° 55.207ʹ N, 23° 32.323ʹ W) and Plateau (*n* = 445 mosquitoes, within 500 m of GPS coordinates 14° 55.371ʹ N, 23° 30.334ʹ W). These two locations are approximately 2.5 km apart. For 62 mosquitoes we could not assign location. Ten BG-Sentinel-2 traps with lure odour bait were placed once a week for 24 h at the selected locations. The collected samples were taken to the University of Jean Piaget laboratory for identification to the species level, and determination of sex and gonadotropic status, using a stereomicroscope and the taxonomic key of mosquitoes in Cabo Verde [16]. After each specimen was identified, all the *Ae. aegypti* mosquitoes collected were individually immersed in 300 µl of RNAlater Stabilization Solution (Invitrogen, Thermo Fisher Scientific, Waltham, MA, USA) and stored at − 20 °C until they were transported to the London School of Hygiene and Tropical Medicine.

### Extraction of DNA and RNA and virus detection

Individual mosquitoes in 1.5 ml microcentrifuge tubes were centrifuged for 2 min at 1300× *rpm* and supernatant was removed. Samples were then washed two times with 1 ml phosphate saline buffer (PBS) and resuspended in 300 ml of RLT buffer containing β-mercaptoethanol (10 μl/ml). Cells were disrupted using Tissue Ruptor II (Qiagen, Hilden, Germany) at speed 3 for 60 s. DNA and RNA of each single mosquito were extracted using Qiagen AllPrep DNA/RNA 96 Kit (Qiagen, Hilden, Germany) following the manufacturer’s protocol. The quantity of DNA in each sample was measured using the Qubit 2.0 fluorimeter HS DNA Kit (Thermo Fisher Scientific, Waltham, MA, USA). The DNA quantity for each sample was variable with < 1 µg obtained per sample. cDNA was synthesized and amplified from RNA using QuantiNova Probe RT-PCR Kit (Qiagen) following the manufacturer’s protocol. Individual mosquitoes were screened using primers and probes to detect the presence of DENV (DENV1-3 and DENV-4) and ZIKV (NS5) (Additional file 1: Table S1). Primers/probes for ZIKV were modified from Grubaugh et al. [7] to take into account the genetic diversity in Asian and American ZIKV samples using data from the NCBI. Each probe contained a fluorescent reporter dye 6-carboxyfluorescein (FAM) at the 5’-end and the Black Hole Quencher 1 at the 3’-end.

### Mosquito *kdr* and *nad*4 sequencing

The primers used to amplify exons 21 and 31 of the *VGSC* gene, which encode for domain II subunit 5 and domain III subunit 6, respectively [31], as well as the mitochondrial *nad*4 gene [35] are described in Additional file 1: Table S2. Polymerase chain reaction (PCR) was carried out in a total volume of 25 µl using standard protocols with 1× reaction buffer, 200 µM dNTP, 0.5 µM forward primer, 0.5 µM reverse primer, 0.02 U/µl DNA polymerase, 17.5 µl water and 1.5 µl DNA sample (1–5 ng). The PCR thermocycler profile consisted of: 1 cycle at 98 °C for 30 s, 30 cycles at 98 °C for 10 s, 56 °C (*VGSC* gene) or 59 °C (*nad*4 gene) for 30 s, 72 °C for 30 s and a final cycle at 72 °C for 2 min. PCR products (10 µl each) were detected by 1% agarose gel electrophoresis in TAE buffer, stained using GelRed (Cambridge Bioscience, Cambridge, UK). The samples were sequenced by capillary sequencing. The resulting DNA sequences have been submitted to GenBank under the accession numbers MT721877-MT721961.

### Mosquito haplotype and phylogenetic analysis

Sequences were trimmed and edited using Geneious (version 11.0) [36]. BLASTn was used to confirm the taxonomic identification. Our *nad*4 sequences were added to a dataset of available *Ae. aegypti nad*4 sequences (*n* = 1101) downloaded from GenBank. Sequences with > 25% missing data and mislabelled as other *Aedes* species were excluded. The sequences were aligned using MAFFT (v7.450). The multiple sequence alignment (MSA) was visualized and trimmed using AliView (v1.23). The trees in Additional file 2: Figure S1 and Additional file 3: Figure S2 were inferred using IQTREE (v1.6.12) with automatic selection of the best-fit. For sequences which had collection date information available, TempEst (v1.5.1) [37] was used to investigate the temporal signal and ‛clocklikenessʼ of our molecular phylogeny. To investigate the genetic diversity of the *Ae. aegypti* mosquitoes, a haplotype network was constructed using the R-package *PEGAS* [38]. Here, *nad*4 sequences from Cape Verde [present study: Tira Chapeu (*n* = 23), Plateau (*n* = 62); other study collected during 2007 and 2010 in City of Praia (*n* = 7) [18] and other countries [NCBI GenBank, total (*n* = 977); Asia (*n* = 607), South America (*n* = 296); North America (*n* = 17); Africa (*n* = 56); and Europe (*n* = 1)] were analysed. Sequences with > 25% missing sequence data were omitted. A neighbour-joining clustering method was chosen to infer the phylogeny [39]. The evolutionary distances were computed using the number of differences method. Branch lengths were inferred using the same units as those of the evolutionary distances. MEGA X (v10.1.6) [40] and FigTree (v1.4.2) software tools were used to manipulate the resulting tree. Haplotype diversity (*h*), nucleotide diversity (*π*) and the neutral mutation Tajima’s *D* were calculated using the R-package *PEGAS*.

## Results

### Prevalence of arbovirus infections

All mosquito samples were DENV negative and two mosquitoes were positive for ZIKV infection (0.25%). The two ZIKV positive *Ae. aegypti* mosquitoes were collected from different locations at different times: (i) 24 August 2016 at Plateau (14° 55.229ʹ N, 23° 30.500ʹ W); and (ii) 5 October 2016 at Tira Chapéu (14° 54.327ʹ N, 23° 31.285ʹ W) (Fig. 1).

### Knockdown resistance (*kdr*) mutations

Two hundred out of 816 *Ae. aeygpti* mosquitoes were selected randomly and screened for *kdr* mutations in exon 21 to check for the V1016G/I and S989P mutations and were also screened for exon 31, to investigate the presence of the F1534C mutation. Sequences were edited, trimmed and poor-quality sequences were excluded from the dataset, resulting in a total of 124 sequences from exon 21 and 133 sequences from exon 31. Nucleotide sequences were compared with 200 or 245 other sequences available in GenBank for exons 21 and 31, respectively. The V1016G/I, S989P and F1534C mutations associated with insecticide resistance were not observed. Furthermore, no other single nucleotide polymorphisms (SNP) were detected.

### Population genetics and phylogenetic analysis of the *nad*4 gene

The alignment of 85 high quality mitochondrial *nad*4 gene sequences (291 bp length) from Cape Verde sourced *Ae. aegypti* mosquitoes revealed the presence of 20 SNPs and 11 haplotypes (5 singletons, i.e. haplotypes found in only one sample each; Table 1). A combined analysis of the Cape Verdean and other countries sequences (*n* = 977) revealed a total of 182 haplotypes, of which 144 were singletons, with a nucleotide diversity (*π*) of 0.0048 and a haplotype diversity (*h*) of 0.71, similar to previous reports in Cape Verde (*π* = 0.002 and *h* = 0.609) [41]. The Tajima’s *D* value was negative (-1.91), but not statistically significant (*P*-value = 0.056).

**Table 1:** Mitochondrial *nad*4 global haplotype frequency in *Aedes aegypti* mosquitoes including the top five most frequent haplotypes per region

Haplotype/Region	Haplotype frequency (%)				
	Africa	Cape Verde (2016)^a^	North America	South America	Asia
XI	20.9	17.3	17.6	1.0	0.3
V	7.0	8.6	0.0	9.8	1.2
XXII	7.0	0.0	5.9	0.0	0.0
XLVI	4.7	0.0	0.0	24.0	42.5
IX	4.7	50.6	0.0	7.1	0.0
XXIX	0.0	0.0	17.6	5.7	0.0
XXXIX	0.0	0.0	5.9	4.7	0.2
XXXIV	0.0	0.0	5.9	0.7	26.9
VI	0.0	1.2	17.6	0.0	0.0
XLVII	0.0	0.0	0.0	0.0	12.4
LXIII	0.0	0.0	0.0	0.0	4.3

^a^Samples collected in this study

**Fig. 2:**
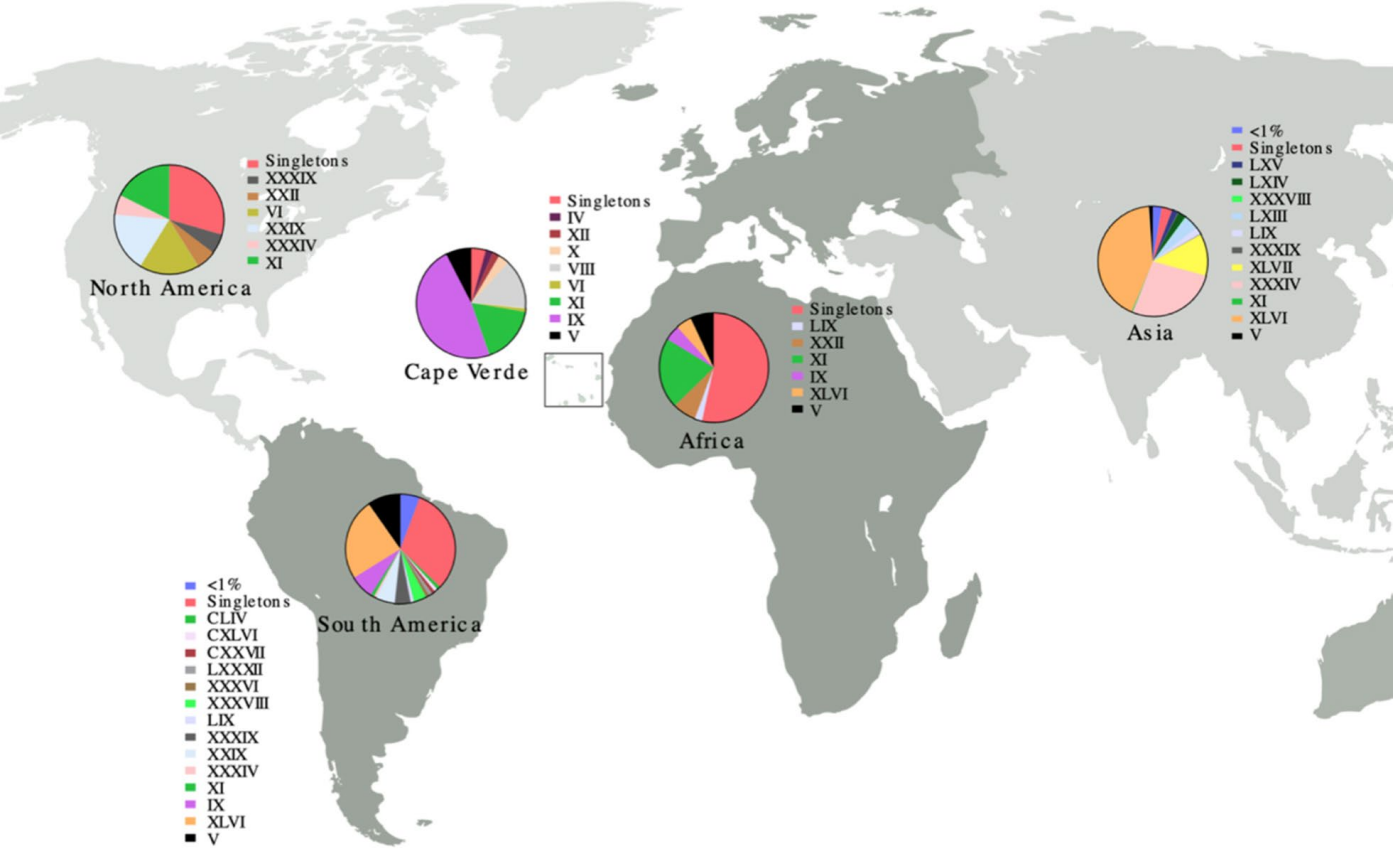
Global map showing the distribution of the *Aedes aegypti* mitochondrial *nad*4 haplotypes. The colours represent the different haplotypes while the size of the pie chart represents the sample size

African samples (*n* = 56) had the highest number of singletons (55.8% of the haplotypes) and the most frequent haplotype (XI, 20.9%) was also present in the other global populations (Fig. 2, Table 1). Asia (*n* = 607) had the lowest frequency of singletons (3.5%) and two main haplotypes. The most frequent haplotype in Asia (XLVI, 42.5%) was also the most frequent in South America (XLVI, *n* = 296, 24.0%) and was present in Africa (XLVI, 4.7%) (Fig. 2, Table 1). The most frequent haplotype in Cape Verde (IX, 50.6%) was also found in Africa and South America (Fig. 2, Table 2). Haplotype XI, present in all populations and the most frequent in Africa, was the second most common (17.3%) in Cape Verde, followed by haplotype VIII (14.8%), which is unique to the country. Other *nad*4 sequences from Cape Verde mosquitoes (*n* = 7) collected during 2007 and 2010 [41] were also included in the analysis and show that these samples share 5 haplotypes with our Cape Verdean samples. The three most frequent haplotypes are the same for both collections and 6 haplotypes were unique from Cape Verde (Table 2).

**Table 2:** Mitochondrial *nad*4 haplotype frequency in *Aedes aegypti* mosquitoes from Cape Verde including all haplotypes identified in Cape Verde

Haplotype/Region	Haplotype frequency (%)					
	Cape Verde (2016)^a^	Cape Verde (2007–2010)^b^	Africa	South America	North America	Asia
IX	50.6	28.6	4.7	7.1	0.0	0.0
XI	17.3	14.3	20.9	1.0	17.6	0.3
VIII	14.8	14.3	0.0	0.0	0.0	0.0
V	8.6	0.0	7.0	9.8	0.0	1.2
I	1.2	0.0	0.0	0.0	0.0	0.0
II	1.2	0.0	0.0	0.0	0.0	0.0
III	1.2	0.0	0.0	0.0	0.0	0.0
IV	1.2	14.3	0.0	0.0	0.0	0.0
VI	1.2	0.0	0.0	0.0	17.6	0.0
VII	1.2	0.0	0.0	0.0	0.0	0.0
X	1.2	14.3	0.0	0.0	0.0	0.0
XII	0.0	14.3	0.0	0.0	0.0	0.0

^a^Samples collected in this study

^b^Samples collected in a previous study [18]

**Fig. 3:**
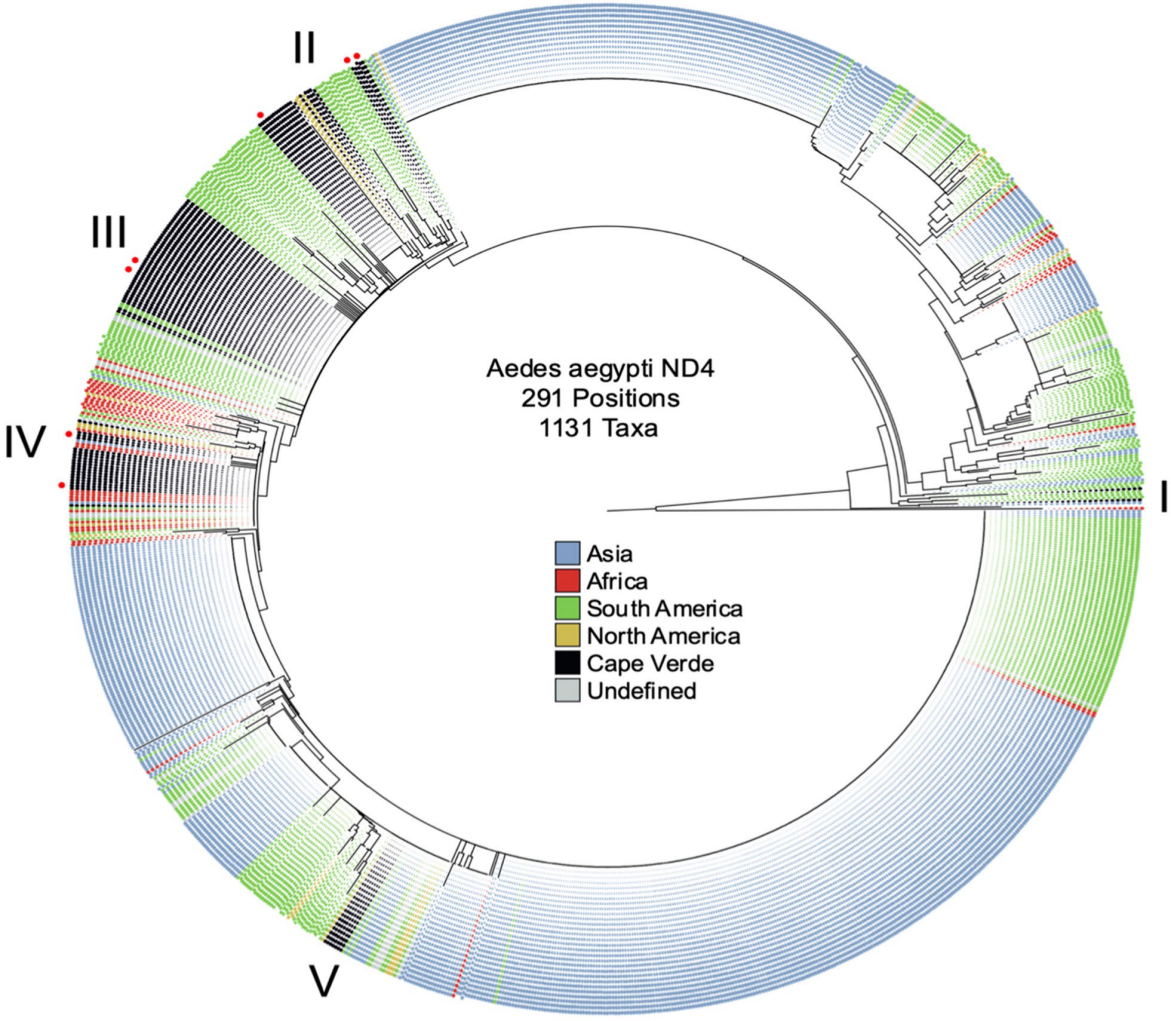
*Aedes aegypti* phylogenetic tree based on the mitochondrial *nad*4 gene. A total of 291 positions were used from 1131 sequences. Clustering was performed using the neighbour joining method. Leaf colour indicates the continental origin. Grey colour indicates undefined geographical origin. Red markers denote Cape Verdean sequences from Salgueiro et al. [20] (CV1-7). Numerals I-V indicate Cape Verdean clusters

A median-joining network, excluding singletons and low frequent haplotypes (< 1%), shows a core haplotype (XI) that includes samples from across all continents and connects with clusters from predominantly Asia and South America (Additional file 4: Figure S3). Using the same dataset, but including all sequences, we constructed a phylogenetic tree (total branch length of 325.06; Fig. 3), which showed that Cape Verde *Ae. aegypti* samples are more related to samples from Africa and South America. Across our 85 Cape Verdean sequences (and 7 previously published), we observed 5 distinct clusters (denoted I to V) with high inter-cluster diversity. Cluster I included 2 Cape Verdean samples and consisted primarily of South American isolates paired with a single Asian isolate. Cluster II included 22 Cape Verdean samples, including 1 pre-existing local isolate and all grouping with sequences from the Americas. Cluster III contained 47% of the Cape Verdean sequences and also included 2 previously described local sequences, 2 other African isolates and 15 South American, all of which shared 97% identity. Cluster IV included 18 Cape Verdean samples, including 2 other publicly available samples. This cluster resided within a predominantly African clade with 75% of the isolates originating from that continent. Cluster V contained 7 Cape Verdean isolates, all of which originate from our study. The rest of the clade consisted of Asian (*n* = 7), African (*n* = 3) and South American (*n* = 7) samples. There was strong clustering of our dataset with the other Cape Verdean previously published sequences, with several having 100% identity. Clusters II and V did not contain any samples from Cape Verde previously reported and therefore are novel undescribed Cape Verdean sequences. No correlation was observed between the Cape Verdean haplotype frequency and sampling site (Additional file 2: Figure S1).

The phylogenetic tree also showed across all samples two major distinct clades, indicating the presence of two separate lineages (Additional file 3: Figure S2) and supporting a previous report [41]. These clades contained isolates from all continents and showed no specific geospatial signal. Using TempEst software, there was weak evidence of a temporal signal across the *nad*4 sequence dataset (R^2^ value of 0.015), not sufficient for the calibration of an accurate molecular clock. However, this analysis would be improved with greater depth of sampling and availability of date of collection, particularly from Africa, where the sequences only represented 4.4% of the total dataset.

## Discussion

The ZIKV outbreak in the Cape Verde archipelago began around October 2015 and lasted until June 2016 with cases mainly reported in in Santiago Island, particularly in Praia, the capital and largest city. We performed an entomological surveillance study to detect the circulation of arboviruses, as this represents a valuable tool to assist in the prevention of further outbreaks, especially in areas with the possible co-circulation of different arboviruses. In our study, we screened 816 *Ae. aegypti* mosquitoes collected in Praia post-outbreak and found a low number of ZIKV-positive (2 in 816) and no DENV-positive mosquitoes. Low levels of ZIKV circulating among mosquito populations post-outbreak have been reported in other countries. For example, post-outbreak ZIKV was detected in pools of *Ae. aegypti* in southern Mexico (15/55 pools of 472 female mosquitoes) and Rio de Janeiro (3/198 pools of 315 female and 235 male mosquitoes) [42, 43]. In Singapore, *Ae. aegypti* mosquitoes were caught during an ongoing outbreak and the number of ZIKV detected in mosquitoes was low (9/1051) [44]. The low level of ZIKV prevalence in *Ae. aegypti* may be due to various factors: the application of strong vector control during and after the outbreak, the timing of sample collection at the end of the outbreak, differences in vector competence of the *Ae. aegypti* from Cape Verde to transmit the ZIKV strain [45], or to a low detection sensitivity of ZIKV due to degradation of viral RNA.

As the use of insecticides represents currently the primary choice for vector control, it is essential to detect insecticide resistance at an early stage to inform control programmes of the most effective measures. This should be done through active surveillance programmes that monitor insecticide resistance through using bioassays or by detecting associated mutations. Knockdown resistance associated with pyrethroid resistance occurs with certain mutations in the *VGSC* gene. Several *kdr* resistance mutations have been identified, with F1534C being the most widely reported [30], but others are geographically distributed, including V1016G mutation in Asia [29], V1016I in Africa and the Americas, [30, 46], and I1011M is found in the Americas [31]. Other mutations have been detected but only a few have been functionally confirmed to confer resistance, including F1534C, V1016G, I1011M and also S989P and the recent V410L [28, 47–49]. In our study, we found that the mosquito samples screened in Cape Verde did not show any of the previously published insecticide resistance mutations such as V1016G/I, S989P and F1534C. No other non-synonymous mutations were detected in these regions of the gene. Two other studies conducted in *Ae. aegypti* from Cape Verde collected between 2007 and 2014 also did not detect any of these mutations [18, 32]. A recent investigation performed in *Anopheles arabiensis* populations in Praia city, revealed the presence of the *kdr* mutation L1014S at a frequency of 7%, and it was suggested that pyrethroid resistance may arise, sweep through the mosquito population, and affect the process of malaria elimination [50]. Although no *kdr* mutations have been detected in the *Ae. aegypti* population, it is possible that the mosquitoes may have acquired metabolic resistance, frequently associated with the overexpression of enzymes responsible for the insecticide detoxification. The absence of co-relation between insecticide resistance and *kdr* allele frequency has been reported in *Anopheles* and *Aedes* mosquitoes suggesting metabolic mechanisms may also contribute to the resistant phenotype [51, 52]. In Cape Verde, bioassay results have previously shown that mosquitoes collected in 2009 were resistant to DDT while mosquitoes collected in 2012 were already resistant to deltamethrin (pyrethroid), cypermethrin (pyrethroid) and also temephos (organophosphate), but susceptible to malathion (organophosphate) [32, 33] The use of temephos and deltamethrin was reinforced in Cape Verde after the DENV (2009) and ZIKV (2016) outbreaks. Investigations of the changes in the frequency of *kdr* mutations in response to insecticide treatments have shown annual increases in mosquito populations across geographical locations. A study of *An*. *gambiae* resistance in Burkina Faso detected a significant increase in *kdr* mutation frequency between 2008 and 2010 [53]. Likewise, a survey of *Ae. aegypti* in Venezuela during 2008, 2010 and 2012 reported that the I1016 allele frequency increased from 0.01 to 0.37, and for C1534 from 0.35 up to fixation, both due to selection effects with deltamethrin [54]. Continuous monitoring of mosquitos across Cape Verde is important to obtain a clearer picture of underlying and changing insecticide resistance profiles in the country, thereby understand the emergence and spread of resistance, and inform vector control programmes.

We have also performed haplotype and phylogenetic analysis using sequence data of the mitochondrial *nad*4 gene from Africa, America and Asia. The analysis indicated that the Cape Verdean *Ae*. *aegypti* are related to those from other countries in Africa and South America, corroborating with historical facts. The origin of the populations of *Ae. aegypti* mosquitoes using mitochondrial genes has been previously investigated. Population genetic analyses conducted with samples from Brazil using the *nad*4 gene among 163 mosquitoes identified two clades sharing haplotypes with populations from West Africa and Asia, and similar results were obtained with the mitochondrial *cytochrome c oxidase subunit 1* (*cox*1) gene [21]. Similar work based on nine microsatellite loci and both *nad*4 and *cox*1 sequences carried out in Bolivia also revealed the existence of two clades, one related with West Africa [55]. Phylogenetic analyses using 34 *nad*4 unique haplotypes from African *Ae. aegypti* mosquitoes and global sequences also revealed 2 clades, with the global haplotypes occurring in both clades [41]. In our results, using a larger dataset of global samples, we also observed two distinct clades contain isolates from all continents, with the Cape Verdean samples located within either clade. A previous study analysing the genetic diversity of samples collected from Cape Verde suggested a West African origin of local mosquitoes [18]. Several of the haplotypes detected in our sample collection were also present in the previously reported Cape Verdean samples. In total, 6 haplotypes were unique to the Cape Verdean mosquitoes while the other haplotypes were present particularly in African and American populations. Historically, *Ae. aegypti* was first detected in 1931 on the island of São Vicente, probably with origin from close African countries. The establishment of trade routes between Europe, Africa and the New World in the 15th century led to the dissemination of *Ae. aegypti* [11, 18]. Following its introduction into the Americas from West Africa *via* slave trade ships between 15th and 18th centuries, *Ae. aegypti* mosquitoes disseminated westwards to the Asia-Pacific region in the late 19th century. Since then, population growth, urbanization and climate change has allowed *Ae. aegypti* to thrive, with suitable foci in 188 countries/territories [1, 15].

History has shown that the opening of travel and trade routes between countries has been accompanied by the spread of mosquitoes and arboviruses, even more so now with the expansion of global air travel. Cape Verde has a strategic location in the middle of the Atlantic, with established air and sea lines and hence heavy traffic coming in and out of the country’s four international airports and four international ports. The economy of Cape Verde relies heavily on tourism and in the importation of goods due to the lack of natural resources. The constant flow of human and trade traffic, alongside with an established *Ae. aegypti* population, means that Cape Verde will always be at risk of arboviral importation. Molecular and epidemiological results indicate that DENV was imported to Cape Verde from Senegal [23] and ZIKV from Brazil [25, 33]. There is a great risk that other vector borne diseases, such as CHIKV and West Nile virus, could be imported into Cape Verde, hence control measures, including strengthening mosquito surveillance, are essential.

## Conclusions

Our results showed that the populations of *Ae. aegypti* collected in Praia at the end of the ZIKV outbreak displayed a low rate of ZIKV infection. In addition*, kdr* mutations associated with insecticide resistance were not detected. Haplotype and phylogeny analysis revealed unique haplotypes in the Cape Verdean *Ae. aegypti* and also indicated that these mosquitoes are related to populations found in Africa and South America. Since Cape Verde has a strategic location with a constant movement of human and trade traffic, studies on vector and pathogen screening, including early detection of insecticide resistance and screening for arboviruses, should be ongoing to support vector control measures and rapid response to future outbreaks in the country.

## Supplementary information

**Additional file 1: Table S1.** Primers and probes used to detect Zika (ZIKV) and dengue (DENV) virus. **Table S2.** *Aedes aegypti* mitochondrial *nad*4 and *VGSC* primers for PCR assays.

**Additional file 2: Figure S1.** Phylogenetic tree inferred using only sequences collected in Cape Verde. The maximum-likelihood phylogeny was inferred using IQTREE with automatic selection of the best-fit model.

**Additional file 3: Figure S2.** Phylogenetic tree using unique haplotypes per country (*n* = 262). Colouration of leaves indicates isolate continental origin. Taxa included in Moore et al.[44] are annotated in blue (clade 1) and red (clade 2). Novel Cape Verdean Sequences presented in this study are indicated (*) alongside pre-existing publicly available isolates (•). The maximum-likelihood tree was inferred using IQTREE with automatic selection of the best-fit model.

**Additional file 4: Figure S3.** Haplotype Network based on *Aedes aegypti* mitochondrial *ND4* sequences. The number of circles represents the number of haplotypes found in Cape Verde. The colours represent the countries in the dataset. The size of the circles does not represent the sample size as 62% of the data are from Asia. The haplotypes are connected by a straight line if they differ by a single mutational step. Singletons and haplotypes with low frequency were not included.

## Data Availability

Data supporting the conclusions of this article are included within the article and its additional files. The newly generated sequences were deposited in the GenBank database under the accession numbers MT721877-MT721961.

## References

[1] Leta S, Beyene TJ, De Clercq EM, Amenu K, Kraemer MUG, Revie CW (2018). Global risk mapping for major diseases transmitted by *Aedes aegypti* and *Aedes albopictus*. Int J Infect Dis..

[2] Weaver SC, Charlier C, Vasilakis N, Lecuit M (2018). Zika, chikungunya, and other emerging vector-borne viral diseases. Annu Rev Med..

[3] Bhatt S, Gething PW, Brady OJ, Messina JP, Farlow AW, Moyes CL (2013). The global distribution and burden of dengue. Nature..

[4] WHO. Factsheet Vector-borne diseases. Geneva: World Health Organization. 2014. http://www.who.int/kobe_centre/mediacentre/vbdfactsheet.pdf.

[5] Faria NR, Quick J, Claro IM, Thézé J, de Jesus JG, Giovanetti M (2017). Establishment and cryptic transmission of Zika virus in Brazil and the Americas. Nature..

[6] Krauer F, Riesen M, Reveiz L, Oladapo OT, Martínez-Vega R, Porgo TV (2017). Zika virus infection as a cause of congenital brain abnormalities and Guillain-Barré syndrome: systematic review. PLoS Med..

[7] Grubaugh ND, Ladner JT, Kraemer MUG, Dudas G, Tan AL, Gangavarapu K (2017). Genomic epidemiology reveals multiple introductions of Zika virus into the United States. Nature..

[8] Patterson J, Sammon M, Garg M (2016). Dengue, Zika and chikungunya: emerging arboviruses in the New World. West J Emerg Med..

[9] Costa-da-Silva AL, Ioshino RS, Petersen V, Lima AF, Cunha M, Wiley MR (2017). First report of naturally infected *Aedes aegypti* with chikungunya virus genotype ECSA in the Americas. PLoS Negl Trop Dis..

[10] Kraemer MUG, Faria NR, Reiner RC, Golding N, Nikolay B, Stasse S (2017). Spread of yellow fever virus outbreak in Angola and the Democratic Republic of the Congo 2015–16: a modelling study. Lancet Infect Dis..

[11] Powell JR, Tabachnick WJ (2013). History of domestication and spread of *Aedes aegypti* - a review. Mem Inst Oswaldo Cruz..

[12] Edman JD, Strickman D, Kittayapong P, Scott TW (1992). Female *Aedes aegypti* (Diptera: Culicidae) in Thailand rarely feed on sugar. J Med Entomol..

[13] Scott TW, Clark GG, Lorenz LH, Amerasinghe PH, Reiter P, Edman JD (1993). Detection of multiple blood feeding in *Aedes aegypti* (Diptera: Culicidae) during a single gonotrophic cycle using a histologic technique. J Med Entomol..

[14] Scott TW, Takken W (2012). Feeding strategies of anthropophilic mosquitoes result in increased risk of pathogen transmission. Trends Parasitol..

[15] Kraemer MUG, Sinka ME, Duda KA, Mylne AQN, Shearer FM, Barker CM (2015). The global distribution of the arbovirus vectors *Aedes aegypti* and *Ae. albopictus*. Elife..

[16] Ribeiro H, da Cunha Ramos H, Capela RA, Pires CA (1980). Os mosquitos de Cabo Verde, sistemática, distribuição, bioecologia, e importância médica.

[17] Alves J, Gomes B, Rodrigues R, Silva J, Arez AP, Pinto J (2010). Mosquito fauna on the Cape Verde Islands (West Africa): an update on species distribution and a new finding. J Vector Ecol..

[18] Salgueiro P, Serrano C, Gomes B, Alves J, Sousa CA, Abecasis A (2019). Phylogeography and invasion history of *Aedes aegypti*, the dengue and Zika mosquito vector in Cape Verde islands (West Africa). Evol Appl..

[19] Gloria-Soria A, Ayala D, Bheecarry A, Calderon-Arguedas O, Chadee DD, Chiappero M (2016). Global genetic diversity of *Aedes aegypti*. Mol Ecol..

[20] Bennett KL, Shija F, Linton YM, Misinzo G, Kaddumukasa M, Djouaka R (2016). Historical environmental change in Africa drives divergence and admixture of *Aedes aegypti* mosquitoes: a precursor to successful worldwide colonization?. Mol Ecol..

[21] Lima RS, Scarpassa VM (2009). Evidence of two lineages of the dengue vector *Aedes aegypti* in the Brazilian Amazon, based on mitochondrial DNA ND4 gene sequences. Genet Mol Biol..

[22] Xue Y, Zhang X, Huang N, Daly A, Gillson CJ, MacArthur DG (2009). Population differentiation as an indicator of recent positive selection in humans: an empirical evaluation. Genetics..

[23] Franco L, Di Caro A, Carletti F, Vapalahti O, Renaudat C, Zeller H (2010). Recent expansion of dengue virus serotype 3 in West Africa. Euro Surveill..

[24] Guedes DRD, Gomes ETB, Paiva MHS, de Melo-Santos MAV, Alves J, Gómez LF (2017). Circulation of DENV2 and DENV4 in *Aedes aegypti* (Diptera: Culicidae) mosquitoes from Praia, Santiago Island. Cabo Verde. J Insect Sci..

[25] Lourenço J, Monteiro ML, Valdez T, Monteiro Rodrigues J, Pybus O, Rodrigues Faria N. Epidemiology of the Zika virus outbreak in the Cabo Verde Islands, West Africa. PLoS Curr. 2018;10. 10.1371/currents.outbreaks.19433b1e4d007451c691f138e1e67e8c PMC5866102 29637009

[26] Direção Nacional da Saúde Programa Integrado de Luta Contra as Doenças Transmitidas por Vetores e Problemas da Saúde Associados ao Meio Ambiente. 2015. https://www.minsaude.gov.cv/index.php/documentosite/paludismo-e-luta-integrada-de-vetores/400-manual-da-luta-integrada-de-vetores-cabo-verde/file.

[27] Vais H, Williamson MS, Devonshire AL, Usherwood PNR (2001). The molecular interactions of pyrethroid insecticides with insect and mammalian sodium channels. Pest Manag Science..

[28] Moyes CL, Vontas J, Martins AJ, Ng LC, Koou SY, Dusfour I (2017). Contemporary status of insecticide resistance in the major *Aedes* vectors of arboviruses infecting humans. PLoS Negl Trop Dis..

[29] Brengues C, Hawkes NJ, Chandre F, McCarroll L, Duchon S, Guillet P (2003). Pyrethroid and DDT cross-resistance in *Aedes aegypti* is correlated with novel mutations in the voltage-gated sodium channel gene. Med Vet Entomol..

[30] Saavedra-Rodriguez K, Urdaneta-Marquez L, Rajatileka S, Moulton M, Flores AE, Fernandez-Salas I (2007). A mutation in the voltage-gated sodium channel gene associated with pyrethroid resistance in Latin American *Aedes aegypti*. Insect Mol Biol..

[31] Martins AJ, Lins RM, Linss JGB, Peixoto AA, Valle D (2009). Voltage-gated sodium channel polymorphism and metabolic resistance in pyrethroid-resistant *Aedes aegypti* from Brazil. Am J Trop Med Hyg..

[32] Rocha HDR, Paiva MHS, Silva NM, de Araújo AP, Camacho DDR, da Moura AJF (2015). Susceptibility profile of *Aedes aegypti* from Santiago Island, Cabo Verde, to insecticides. Acta Trop..

[33] Dia I, Diagne CT, Ba Y, Diallo D, Konate L, Diallo M (2012). Insecticide susceptibility of *Aedes aegypti* populations from Senegal and Cape Verde Archipelago. Parasit Vectors..

[34] The dengue vaccine dilemma (2018). Lancet Infec Dis..

[35] Seixas G, Salgueiro P, Silva AC, Campos M, Spenassatto C, Reyes-Lugo M (2013). *Aedes aegypti* on Madeira Island (Portugal): genetic variation of a recently introduced dengue vector. Mem Inst Oswaldo Cruz..

[36] Kearse M, Moir R, Wilson A, Stones-Havas S, Cheung M, Sturrock S (2012). Geneious Basic: an integrated and extendable desktop software platform for the organization and analysis of sequence data. Bioinformatics..

[37] Rambaut A, Lam TT, Carvalho LM, Pybus OG. Exploring the temporal structure of heterochronous sequences using TempEst (formerly Path-O-Gen). Virus Evol. 2016;2:vew007. 10.1093/ve/vew007 PMC4989882 27774300

[38] Paradis E (2010). Pegas: an R package for population genetics with an integrated-modular approach. Bioinformatics..

[39] Saitou N, Nei M (1987). The neighbor-joining method: a new method for reconstructing phylogenetic trees. Mol Biol Evol..

[40] Kumar S, Stecher G, Li M, Knyaz C, Tamura K (2018). MEGAX: Molecular Evolutionary Genetics Analysis across computing platforms. Mol Biol Evol..

[41] Moore M, Sylla M, Goss L, Burugu MW, Sang R, Kamau LW (2013). Dual African origins of global *Aedes aegypti sl* populations revealed by mitochondrial DNA. PLoS Negl Trop Dis..

[42] Guerbois M, Fernandez-Salas I, Azar SR, Danis-Lozano R, Alpuche-Aranda CM, Leal G (2016). Outbreak of Zika virus infection, Chiapas State, Mexico, 2015, and first confirmed transmission by *Aedes aegypti* mosquitoes in the Americas. J Infect Dis..

[43] Ferreira-de-Brito A, Ribeiro IP, Miranda RM, Fernandes RS, Campos SS, Silva KAB (2016). First detection of natural infection of *Aedes aegypt*i with Zika virus in Brazil and throughout South America. Mem Inst Oswaldo Cruz..

[44] Ho ZJM, Hapuarachchi HC, Barkham T, Chow A, Ng LC, Lee JMV (2017). Outbreak of Zika virus infection in Singapore: an epidemiological, entomological, virological, and clinical analysis. Lancet Infect Dis..

[45] Calvez E, O’Connor O, Pol M, Rousset D, Faye O, Richard V (2018). Differential transmission of Asian and African Zika virus lineages by *Aedes aegypti* from New Caledonia. Emerg Microbes Infect..

[46] Zardkoohi A, Castañeda D, Lol JC, Castillo C, Lopez F, Rodriguez RM, Padilla N (2020). Co-occurrence of kdr Mutations V1016I and F1534C and Its Association With Phenotypic Resistance to Pyrethroids in Aedes aegypti (Diptera: Culicidae) Populations From Costa Rica. J Med Entomol..

[47] Sombié A, Saiki E, Yaméogo F, Sakurai T, Shirozu T, Fukumoto S (2019). High frequencies of F1534C and V1016I *kdr* mutations and association with pyrethroid resistance in *Aedes aegypti* from Somgandé (Ouagadougou). Burkina Faso. Trop Med Health..

[48] Haddi K, Tomé HVV, Du Y, Valbon WR, Nomura Y, Martins GF (2017). Detection of a new pyrethroid resistance mutation (V410L) in the sodium channel of *Aedes aegypti*: a potential challenge for mosquito control. Sci Rep..

[49] Du Y, Nomura Y, Satar G, Hu Z, Nauen R, He SY (2013). Molecular evidence for dual pyrethroid-receptor sites on a mosquito sodium channel. Proc Natl Acad Sci USA.

[50] da Cruz DL, Paiva MHS, Guedes DRD, Alves J, Gómez LF, Ayres CFJ (2019). Detection of alleles associated with resistance to chemical insecticide in the malaria vector *Anopheles arabiensis* in Santiago. Cabo Verde. Malar J..

[51] Messenger LA, Shililu J, Irish SR, Anshebo GY, Tesfaye AG, Ye-Ebiyo Y (2017). Insecticide resistance in *Anopheles arabiensis* from Ethiopia (2012–2016): a nationwide study for insecticide resistance monitoring. Malar J..

[52] Ngoagouni C, Kamgang B, Brengues C, Yahouedo G, Paupy C, Nakouné E (2016). Susceptibility profile and metabolic mechanisms involved in *Aedes aegypti* and *Aedes albopictus* resistant to DDT and deltamethrin in the Central African Republic. Parasit Vectors..

[53] Badolo A, Traore A, Jones CM, Sanou A, Flood L, Guelbeogo WM (2012). Three years of insecticide resistance monitoring in *Anopheles gambiae* in Burkina Faso: resistance on the rise?. Malar J..

[54] Alvarez LC, Ponce G, Saavedra-Rodriguez K, Lopez B, Flores AE (2015). Frequency of V1016I and F1534C mutations in the voltage-gated sodium channel gene in *Aedes aegypti* in Venezuela. Pest Manag Sci..

[55] Paupy C, Le Goff G, Brengues C, Guerra M, Revollo J, Barja Simon Z (2012). Genetic structure and phylogeography of *Aedes aegypti*, the dengue and yellow-fever mosquito vector in Bolivia. Infect Genet Evol..

