# Supplementary material for: Surveillance of Aedes aegypti populations in the city of Praia, Cape Verde: Zika virus infection, insecticide resistance and genetic diversity
Source: Parasit Vectors. 2020 Sep 21;13:481. doi: 10.1186/s13071-020-04356-z (PMC7507728; doi:10.1186/s13071-020-04356-z)
Supplement: Supplementary file 1 — Additional file 1: Table S1. Primers and probes used to detect Zika (ZIKV) and dengue (DENV) virus. Table S2. Aedes aegypti mitochondrial nad4 and VGSC primers for PCR assays. [file 13071_2020_4356_MOESM1_ESM.docx]

**Additional file 1: Table S1. Primers and probes used to detect Zika (ZIKV) and Dengue (DENV) virus.**

In the sequences Y refers to C or T and R refers to A or G.

| **Virus** | **Forward** | **Reverse** | **Probe** |
| --- | --- | --- | --- |
| ZIKV | AGTGCCAGAGYTGTGTGTAC | TCTAGCCCCTAGCCACATRT | FAM-5′-CAGCCGCGCCATCTGGT- BHQ1 |
| DENV | GGATAGACCAGAGATCCTGCTGT | CATTCCATTTTCTGGCGTTC (DENV1-3)  CAATCCATCTTGCGGCGCTC (DENV4) | CAGCATCATTCCAGGCACAG |

**Additional file 1: Table S2. *Aedes aegypti* Mitochondrial *ND4* and *VGSC* primers for PCR assays**. Table 2 displays the primers sequences used to amplify the mitochondrial *ND4* gene (Mt-ND4) and the partial sequences of the voltage gate sodium channel gene (*VGSC*), exons 21 and 31.

| **Gene** | **Forward** | **Reverse** |
| --- | --- | --- |
| **Mt-ND4** | TGATTGCCTAAGGCTCATGT | TTCGGCTTCCTAGTCGTTCAT |
| **Exon 21** | ACAATGTGGATCGCTTCCC | TGGACAAAAGCAAGGCTAAG |
| **Exon 31** | GACTCGCGGGAGGTAAGTT | CCGTCTGCTTGTAGTGATCG |
