# Supplementary material for: Surveillance of Aedes aegypti populations in the city of Praia, Cape Verde: Zika virus infection, insecticide resistance and genetic diversity
Source: Parasit Vectors. 2020 Sep 21;13:481. doi: 10.1186/s13071-020-04356-z (PMC7507728; doi:10.1186/s13071-020-04356-z)
Supplement: Supplementary file 2 — Additional file 2: Figure S1. Phylogenetic tree inferred using only sequences collected in Cape Verde. The maximum-likelihood phylogeny was inferred using IQTREE with automatic selection of the best-fit model. [file 13071_2020_4356_MOESM2_ESM.pdf]

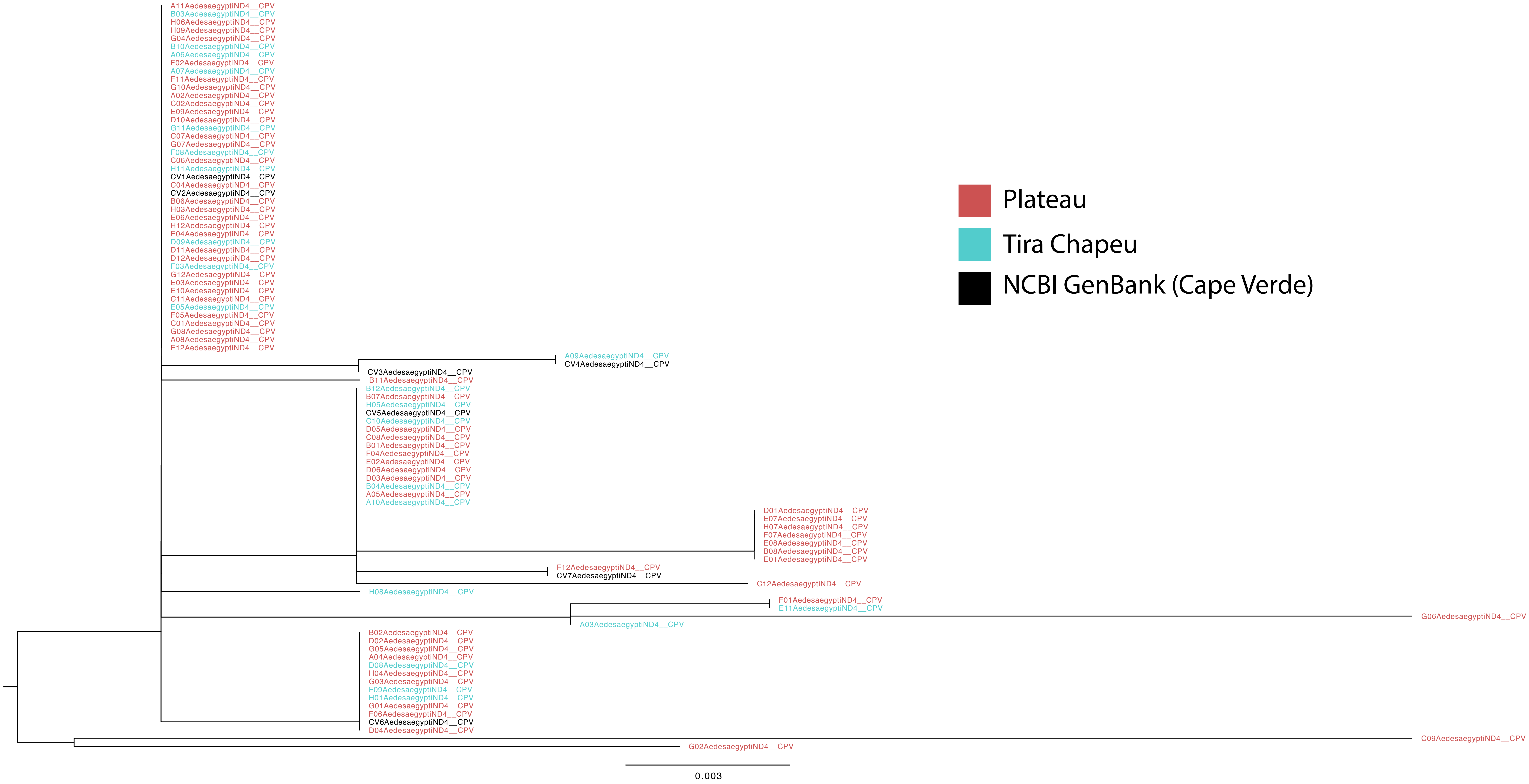
Plateau
Tira Chapeu
NCBI GenBank (Cape Verde)
A11AedesaegyptiND4__CPV
B03AedesaegyptiND4__CPV
H06AedesaegyptiND4__CPV
H09AedesaegyptiND4__CPV
G04AedesaegyptiND4__CPV
B10AedesaegyptiND4__CPV
A06AedesaegyptiND4__CPV
F02AedesaegyptiND4__CPV
A07AedesaegyptiND4__CPV
F11AedesaegyptiND4__CPV
G10AedesaegyptiND4__CPV
A02AedesaegyptiND4__CPV
C02AedesaegyptiND4__CPV
E09AedesaegyptiND4__CPV
D10AedesaegyptiND4__CPV
G11AedesaegyptiND4__CPV
C07AedesaegyptiND4__CPV
G07AedesaegyptiND4__CPV
F08AedesaegyptiND4__CPV
C06AedesaegyptiND4__CPV
H11AedesaegyptiND4__CPV
CV1AedesaegyptiND4__CPV
C04AedesaegyptiND4__CPV
CV2AedesaegyptiND4__CPV
B06AedesaegyptiND4__CPV
H03AedesaegyptiND4__CPV
E06AedesaegyptiND4__CPV
H12AedesaegyptiND4__CPV
E04AedesaegyptiND4__CPV
D09AedesaegyptiND4__CPV
D11AedesaegyptiND4__CPV
D12AedesaegyptiND4__CPV
F03AedesaegyptiND4__CPV
G12AedesaegyptiND4__CPV
E03AedesaegyptiND4__CPV
E10AedesaegyptiND4__CPV
C11AedesaegyptiND4__CPV
E05AedesaegyptiND4__CPV
F05AedesaegyptiND4__CPV
C01AedesaegyptiND4__CPV
G08AedesaegyptiND4__CPV
A08AedesaegyptiND4__CPV
E12AedesaegyptiND4__CPV
A09AedesaegyptiND4__CPV
CV4AedesaegyptiND4__CPV
CV3AedesaegyptiND4__CPV
B11AedesaegyptiND4__CPV
B12AedesaegyptiND4__CPV
B07AedesaegyptiND4__CPV
H05AedesaegyptiND4__CPV
CV5AedesaegyptiND4__CPV
C10AedesaegyptiND4__CPV
D05AedesaegyptiND4__CPV
C08AedesaegyptiND4__CPV
B01AedesaegyptiND4__CPV
F04AedesaegyptiND4__CPV
E02AedesaegyptiND4__CPV
D06AedesaegyptiND4__CPV
D03AedesaegyptiND4__CPV
B04AedesaegyptiND4__CPV
A05AedesaegyptiND4__CPV
A10AedesaegyptiND4__CPV
D01AedesaegyptiND4__CPV
E07AedesaegyptiND4__CPV
H07AedesaegyptiND4__CPV
F07AedesaegyptiND4__CPV
E08AedesaegyptiND4__CPV
B08AedesaegyptiND4__CPV
E01AedesaegyptiND4__CPV
F12AedesaegyptiND4__CPV
CV7AedesaegyptiND4__CPV
C12AedesaegyptiND4__CPV
H08AedesaegyptiND4__CPV
F01AedesaegyptiND4__CPV
E11AedesaegyptiND4__CPV
G06AedesaegyptiND4__CPV
A03AedesaegyptiND4__CPV
B02AedesaegyptiND4__CPV
D02AedesaegyptiND4__CPV
G05AedesaegyptiND4__CPV
A04AedesaegyptiND4__CPV
D08AedesaegyptiND4__CPV
H04AedesaegyptiND4__CPV
G03AedesaegyptiND4__CPV
F09AedesaegyptiND4__CPV
H01AedesaegyptiND4__CPV
G01AedesaegyptiND4__CPV
F06AedesaegyptiND4__CPV
CV6AedesaegyptiND4__CPV
D04AedesaegyptiND4__CPV
C09AedesaegyptiND4__CPV
G02AedesaegyptiND4__CPV
0.003
