# Supplementary figures and images for: Surveillance of Aedes aegypti populations in the city of Praia, Cape Verde: Zika virus infection, insecticide resistance and genetic diversity
Source: Parasit Vectors. 2020 Sep 21;13:481. doi: 10.1186/s13071-020-04356-z (PMC7507728; doi:10.1186/s13071-020-04356-z)

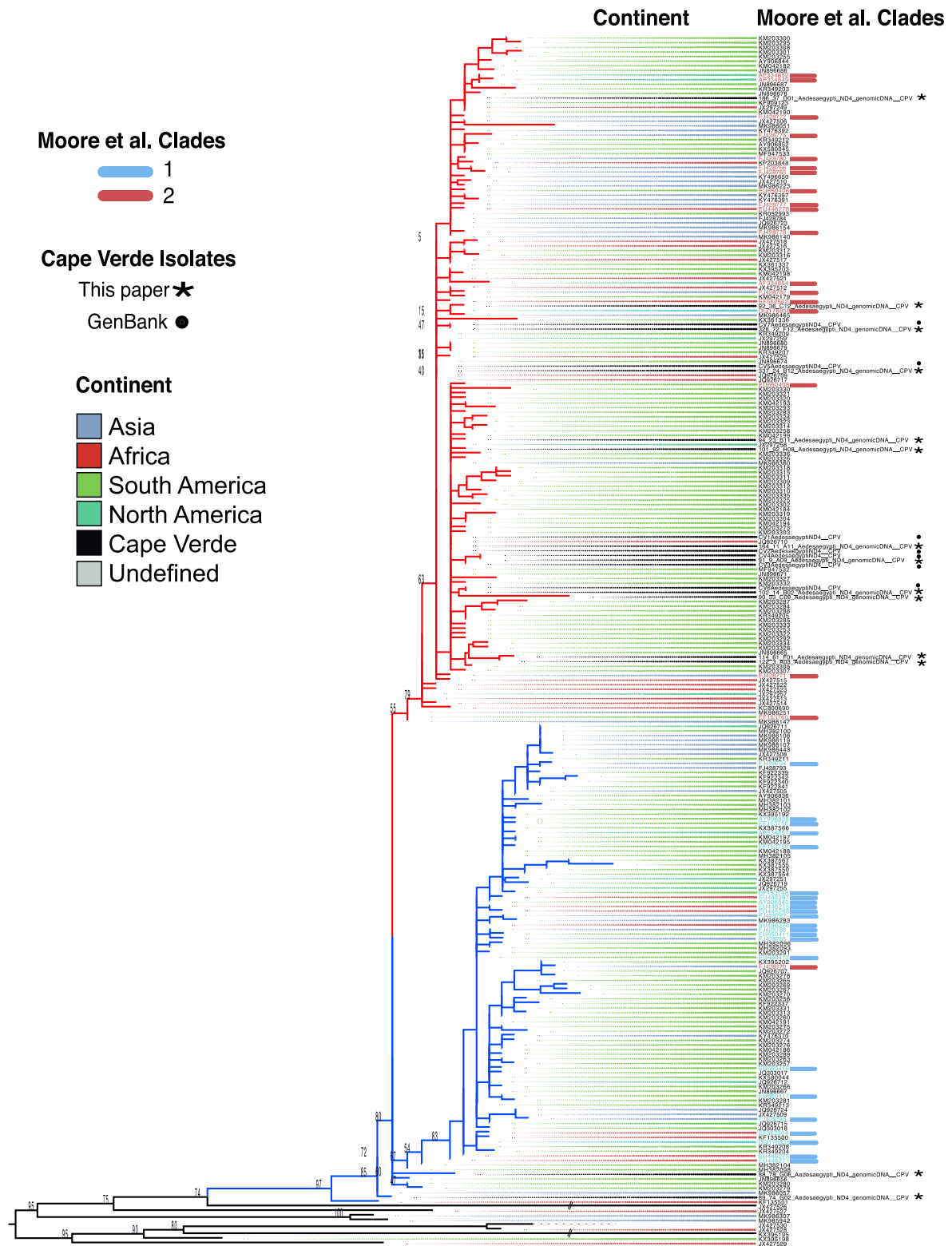

Supplement: Supplementary file 3 — Additional file 3: Figure S2. Phylogenetic tree using unique haplotypes per country (n = 262). Colouration of leaves indicates isolate continental origin. Taxa included in Moore et al.[44] are annotated in blue (clade 1) and red (clade 2). Novel Cape Verdean Sequences presented in this study are indicated (*) alongside pre-existing publicly available isolates (•). The maximum-likelihood tree was inferred using IQTREE with automatic selection of the best-fit model. [file 13071_2020_4356_MOESM3_ESM.pdf]

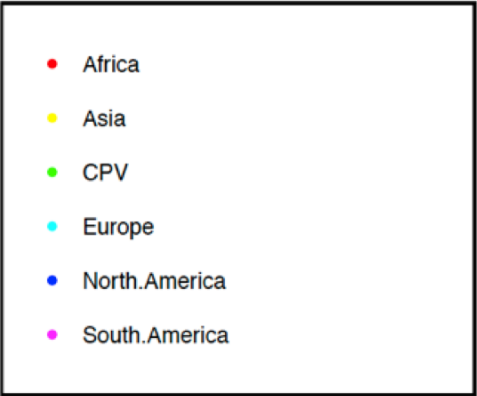

Supplement: Supplementary file 4 — Additional file 4: Figure S3. Haplotype Network based on Aedes aegypti mitochondrial ND4 sequences. The number of circles represents the number of haplotypes found in Cape Verde. The colours represent the countries in the dataset. The size of the circles does not represent the sample size as 62% of the data are from Asia. The haplotypes are connected by a straight line if they differ by a single mutational step. Singletons and haplotypes with low frequency were not included. [file 13071_2020_4356_MOESM4_ESM.pdf]
